# Comments on the Extraperitoneal Approach for Standard Laparoscopic Radical Prostatectomy: What Is Gained and What Is Lost

**DOI:** 10.1155/2011/150978

**Published:** 2011-09-22

**Authors:** Evangelos Liatsikos, Iason Kyriazis, Panagiotis Kallidonis, Minh Do, Tim Haefner, Anja Dietel, Sigrun Holze, Narasimhan Ragavan, Jens-Uwe Stolzenburg

**Affiliations:** ^1^Department of Urology, University of Patras, Medical School, Rion, 26 500 Patras, Greece; ^2^Department of Urology, University of Leipzig, 04109 Leipzig, Germany; ^3^Department of Urology, Royal Liverpool University Teaching Hospitals, L7 8XP Liverpool, UK

## Abstract

Laparoscopic extraperitoneal radical prostatectomy (LERP) is considered the standard care treatment option for the management of localized and locally advanced prostatic cancer (PCa) in many institutes worldwide. In this work, the main advantages and disadvantages of LERP approach are reviewed with regard to its outcomes, the complication management, the learning curve, and the extend of pelvic lymph node dissection (PLND). It is concluded that LERP demonstrates comparable cancer control, urinary continence, and potency outcomes with the open and the robot-assisted radical prostatectomy, while offering advantages in complication management in comparison to the transperitoneal approach. Learning curve of LERP is considered long and stiff and significantly affects perioperative outcomes and morbidity, cancer control, and functional results. Thus, close mentoring especially in the beginning of the learning curve is advised. Finally, LERP still has a role in the limited or modified PLND offered in intermediate risk PCa patients.

## 1. Introduction

Since its introduction in 1992 by Schuessler et al. laparoscopic radical prostatectomy (LRP) has become a standard care for the management of localized and locally advanced prostatic cancer (PCa) in many institutes worldwide [1]. Mimicking conventional open technique, laparoscopy combines similar to open radical prostatectomy trifecta outcomes (cancer control, urinary continence, and potency) with less blood loss and superior cosmesis [2, 3]. Extraperitoneal LRP (LERP), firstly introduced in 1997 by Raboy et al. has been adopted by our departments as the method of choice for radical prostatectomy [4, 5]. In this work, we review the main advantages and disadvantages of LERP approach in comparison with the open, the laparoscopic transperitoneal and the robot-assisted radical prostatectomy.

## 2. Outcomes of LERP

Due to the minimal invasive nature of the laparoscopic technique, LERP is associated with favorable perioperative outcomes. Blood loss in comparison to open approach is minimum rarely requiring transfusion. In our previously reported series of 2400 LERP cases, mean blood loss was 255 mL (range 20–1200) and transfusion rate was 0.7% [5]. Additionally, in experienced hands convention to open surgery is uncommon given that even the most significant complications can be safely managed laparoscopically. The only disadvantage of the laparoscopic approach is that operation times are regularly reported to be longer than open approach [2]. Still, our series had a mean operation time of nearly 2.5 hours indicating that at the end of the learning curve, operation times can be compared favorably with the rest of radical prostatectomy techniques [5].

In matters of the trifecta outcomes, LERP is associated with comparable results with the reference standard open approach. Positive margin rates (PMRs) vary between 8% and 20% for pT_2_ disease and from 30% to 69% for pT_3_ [6]. In our series, positive surgical margins were found in 8% and 35.6% of pT_2_ and pT_3_ cases, which are consistent with the outcomes reported from other high-volume centers concerning open, laparoscopic, or robot-assisted approaches for radical prostatectomy [2, 5, 7]. Additionally, in our series, early urinary continence was evident in 71.7% of patients at three months after LERP and reached 94.7% within one year. Incontinence (more than 2 pads per day) after 1 postoperative year was observed in only 1.3% of our cases. Finally, potency, during the first postoperative year, was reported by 44% and 72% of our patients subjected to unilateral and bilateral nerve sparing LERP accordingly [5].

Direct comparison of open versus laparoscopic versus robot-assisted radical prostatectomy in a prospective randomized setting is lucking. Thus, definite conclusion regarding the superiority of one technique over the others cannot be drawn. Grossi et al. in a case-control, single institution study with a followup up to 7 years, reviewed the outcomes of 50 patients treated via open retropubic prostatectomy with 50 patients subjected to LERP. No significant differences between the two techniques were observed in terms of oncological results within a mean followup of 24 months. Still, LERP was associated with shorter catheterization, recover of continence and potency, shorter hospital stay, and lower transfusion rates [8]. Similarly, McCullough et al. reviewing the morbidity encounter during 96 LERP and 184 open radical prostatectomies, reported that LERP was associated with shorter catheterization time and hospitalization as well as fewer urinary tract infections [9]. In contrast, Memorial Sloan Kettering Cancer Center experience comparing 257 LRP patients with 298 open approaches revealed inferior continence rates (defined as no pads used) in LRP patients [10]. Additionally, Touijer et al. in a nonrandomized prospective study evaluating 612 laparoscopic and 818 open radical prostatectomies revealed that open and laparoscopic approaches had similar oncological outcomes (PMRs and freedom from progression within a mean followup of 18 months), but postoperative visits, readmission rate, and continence were inferior in the laparoscopic approach. Recovery of potency was equivalent between the two techniques, while blood loss and transfusion rates favored the laparoscopic technique [11]. The impact of the long lasting learning curve of laparoscopic approach should be taken into consideration for the interpretation of the discrepancies of functional and oncological outcomes between studies. Data obtained from high-volume centers fail to demonstrate any differences in the trifecta, and consequently in experienced hands both open and laparoscopic approach should be considered equivalent treatment options for PCa [2]. 

Few studies have compared transperitoneal versus extraperitoneal approach for LRP. Cathelineau et al. reviewing 200 consecutives radical prostatectomies performed via a transperitoneal or and extraperitoneal route, did not reveal any differences in terms of operative, postoperative, and pathological data and concluded that each surgeon has to choose considering personal experience, training, and standardization [12]. In contrast, Eden et al. in a similar study excluding the initial cases from each group (considered to be within the learning curve of each technique), revealed superiority for LERP over the transperitoneal approach with respect to operative time, hospitalization and early continence [13]. Similarly, Cohen et al. evaluated retrospectively 265 patients subjected to either transperitoneal or extraperitoneal radical prostatectomy by one surgeon. Improved surgical outcome for the extraperitoneal approach was reveled in regard to operative time, analgesic use, length of hospitalization, urinary leakage, and complication rate. Yet the particular study had several limitations including non grading of complication's severity and that different approaches were not randomly distributed within study period. The extraperitoneal approaches (*n* = 172) followed the 93 transperitoneal approaches, and consequently, surgeon's expert should have been significantly advanced over time leading to superior results [14]. Recently, Siqueira Jr. et al. evaluating the complication of the two approaches during the learning curve (initial 40 cases for each approach), did not reveal any differences in overall complication rates. Yet complications encountered during the transperitoneal approach were judged as more serious due to the potential chance of intraperitoneal peritonitis not observed with the extraperitoneal approach [15].

The main advantage of robotic assistance in LRP over standard laparoscopic approach is the shorter learning curve of the first [16]. Due to the lack of large randomized studies comparing the two approaches, definite conclusions cannot be drawn. Yet a trend of robot-assisted LRP towards better outcomes is evident. Meta-analysis of data obtained by high-volume studies (more than 250 patients) revealed higher continence (92% versus 83.3%) and potency rates after robot-assisted radical prostatectomy versus standard LRP as well as lower weighted mean PSM rates (13.6% versus 21.3%) [2]. The only available randomized controlled trial with 12 months of followup has been recently reported by Asimakopoulos et al. In this study, 64 patients subjected to LRP were compared with 64 patients subjected to robotic-assisted LRP with regard to operating time, estimated blood loss, transfusion rate, complications, rates of positive surgical margins, rates of biochemical recurrence, continence, and time to continence as well as erectile function. No statistically significant differences were observed for all measured parameters apart from the 12-month capability for intercourse (with or without phosphodiesterase type 5 inhibitors) which was significantly higher in the robotic group. Time to capability for intercourse was significantly shorter as well [17]. Larger prospective randomized studies should be expected in the future to elucidate the debate over the superiority of one technique over the other. Additionally, long-term oncological outcomes, currently lacking due to the relative recent worldwide adaptation of these minimally invasive approaches, are necessary in order to clarify the cancer control effectiveness of each case. In contrast, the main disadvantage of robotic-assisted LRP is the significantly higher cost. The latter limits its availability into only high-volume centers and consists of robotic assistance superfluous for the majority of institutes in the developing countries. 

## 3. Complication Management in LERP

We have previously reviewed the identification, management, and prevention of the most common complications encountered during LERP [18]. In general, perioperative complications almost entirely include bleeding and intra-abdominal organ injuries, while ileus, anastomotic leakage or strictures, lymphocele formation, urinary tract infection, and temporary obturator nerve apraxia are the most common postoperative complications. Complications associated with LERP are much associated with surgical experience, given that, as experience expands, the occurrence of complication declines. Comparative assessment of complications reported by high-volume centers between open, laparoscopic, or robot-assisted radical prostatectomy revealed a weighted mean postoperative complication rate around 10%, similar for all approaches [2, 19].

In case of bleeding, laparoscopic and robot-assisted radical prostatectomy demonstrate significantly less blood loss than open approach [2]. The most common sites of vascular injury are the inferior epigastric vessels, the external iliac vein, and the Santorini's venous plexus. No advantages in blood loss between transperitoneal and extraperitoneal LRP exist. Yet inferior epigastric vessel injury is more easily managed extraperitoneally, given that bleeding vessel can be directly traced and coagulated or clipped. It should be emphasized that in case of laparoscopic approaches, intra-abdominal pressure created by gas insufflation tends to perioperatively tamponade venous bleeding from small vessels, which in turn might rarely lead to postoperative preperitoneal of perineal hematoma formation. Conservative management is effective in the majority of such incidences, while laparoscopic or open surgical revision is necessary only in 0.6% of cases [5]. 

The occurrence of persistent ileus in extraperitoneal approach is less likely to occur due to minimum bowel manipulation and the lack of intraperitoneal drainage of leaking fluids (e.g., urine extravasation). Intrabdominal organ injury involves mainly bowel injury and injury of the lower urinary tract (bladder/ureters). Bowel injury can be induced either by accidental thermal injury during coagulation of closely associated structures or by direct perforation. [20, 21]. Extraperitoneal approach consists of accidental thermal injuries quite uncommon due to peritoneal sequestering of bowels outside the operating field. Moreover, bowel injuries, rarely occurring during trocar insertion and dorsal dissection of the overlying rectum prostatic apex, are considered a less severe complication in the extraperitoneal access than the transperitoneal approach, since the risk of generalized peritonitis is diminished [15]. Urinary bladder injury is mostly encountered during the initial dissection of the extraperitoneal space. Thus, previous abdominal wall mesh-hernia repair which is associated with dence bladder-abdominal wall adhesions can be considered a relative contraindication for the extraperitoneal approach [21]. Finally, ureteral injury is uncommon during LERP since ureters can be easily identified and protected. In contrast, 75% of ureteral injuries in cases of transperitoneal approach occur during the posterior dissection of the vesiculodeferential junction or the lateral vesical peritoneum, which is avoided by the extraperitoneal approach [22]. Ureteral orifices are at risk of damage during posterior bladder neck dissection or anastomosis equally in all open or laparoscopic approaches [18]. 

Postoperative anastomotic leakage is one of the most common postoperative complications of LERP occurring in 2.6% of prostatectomies. In case of such complication, extraperitoneal approach is considered beneficial for the healing process not only due to the separation of extravasated urine from the peritoneal cavity but also because upon removal of the insufflation pressure, bladder and perivesical fat fall back tightly around the anastomosis diminishing postoperative extravasation. The formation of a significant postoperative extraperitoneal pelvic hematoma, although rare, can increase the traction on the anastomosis and in some occasions can even leak through the anastomosis. In this situation, catheterisation time must be expanded. Finally, lymphocele formation is a complication encountered in 3.6% of PLNDs via the extraperitoneal route. Higher rates of lymphocele formation in the extraperitoneal access due to the lack of intraperitoneal drainage are considered a limitation of the approach when PLND is initiated. Still, bilateral peritoneal fenestration at the end of LERP in our series significantly reduced the incidence of lymphocele formation [23].

## 4. Learning Curve

LERP is considered a technically challenging approach requiring advance laparoscopic skills, and thus, learning curve of the technique is considered long and stiff. Starling et al. revealed significant differences in urinary incontinence, transfusion, and complications rates between the first 70 and the last 200 LERPs [24]. Similarly, Hruza et al. in a recent evaluation of complication rates during 2200 endoscopic radical prostatectomies (871 transperitoneal and 1329 extraperitoneal), revealed a significant decrease in overall complication rates over time. Interestingly, learning curves of third-generation surgeons were found to be shorter compared to the first generation (250 versus 700 cases) [25]. Finally, Rodriguez et al. demonstrated that learning curve of LERP is not only associated with perioperative outcomes, but with oncologic outcomes as well. While perioperative outcomes improved after 100 cases, significant differences in positive margin rates required 200 cases to be apparent. In addition, positive margin rates kept decreasing even after 300 cases [26]. Rozet et al. in a series of 100 consecutive cases of LERP, reported about half of cases with positive margins occurring within the first 25 patients, while Vickers et al. in a retrospective cohort study, verified that learning curve of LERP is long demonstrating that the benefit of experience in reducing cancer risk continued up to 750 cases [21, 27]. 

As evidenced above, surgical experience in LERP significantly affects both perioperative (operation time, blood loss, and complication rates) and oncologic outcomes. The longer learning curve of LERP than open and robot-assisted prostatectomy should be considered a limitation of the approach during the installation of the technique. Yet the well-documented advantages of laparoscopy over open approach and the relative worldwide unavailability of robot assistance when balanced over the stiff learning curve of laparoscopic approach inevitably lead to the same conclusion. Laparoscopic prostatectomy is becoming the worldwide standard of care for radical prostatectomy, and all available measures to bypass learning curve without compromising patient's safety and oncologic outcome should be taken. Close supervision by experts and modular training programs can significantly shorten learning curve and offer acceptable perioperative and oncologic outcomes in the initial patient series subjected to this highly complex urological procedure [28].

## 5. Pelvic Lymph Node Dissection during LERP

Pelvic lymph node dissection (PLND) is indicated for the staging of high-risk prostatic cancer during radical prostatectomy. A limitation of the extraperitoneal route is that only limited or modified PLND can be performed via this approach and not extended PLND. This is due to the fact that balloon dilation and gas insufflations in the extraperitoneal space cannot uncover the lymph nodes located above the bifurcation of common iliac vessels. Accordingly, advocators of the extraperitoneal technique, in accordance to the trend that when PLND should take place then the extended dissection should be performed, support that in the case of concomitant PLND a transperitoneal approach should be preferred. Indeed, a significant portion of lymph node metastases occur at nodes located outside the resection template of limited PLND, and consequently the latter procedure would underestimate the oncologic staging [29, 30]. Additionally, from an oncologic prospect of view, PLND if done properly should offer a partial decrease of tumor spreading which might improve prognosis. 

Yet the potential curative effect of PLND has not been proved with certainty. Examination of large series of patients with varied risk for LN metastases has failed to prove that PLND alters significantly the biochemical relapse-free survival of the disease [31–33]. The latter could possibly be explained by the fact that even extended PLND fails to retrieve the entire lymph node network draining from the prostate, and as a result, the possibility through a radical prostatectomy and a combining extensive LN resection to extract all spreading cancer cells is very limited. Additionally, Briganti et al. presenting one of the most accurate nomograms, demonstrated that patients with PSA <10 and biopsy Gleason sum less than 7 in a clinical stage T1c or T2 have a very limited (1 and 1.5% accordingly) possibility to harvest exclusively nonobturator LN metastases [30]. Consequently, in this group, which represents the majority of patients subjected to radical prostatectomy, a limited to obturator fosse PLND would have had a quite acceptable accuracy in predicting the true lymph node invasion, and extended PLND should have been avoided. In accordance to the above, extraperitoneal approach still has a place in the treatment of intermediate risk prostate cancer patients requiring PLND.

## 6. The Future of Extraperitoneal Approach

Robotic technology significantly enhances laparoscopy combining superior instrument manipulation, operative field visualization, and convenience for the surgeon, explaining the trend towards replacement of conventional laparoscopy with robot assistance wherever robotic technology is available. As this novel technology is maturing, equipment-purchasing prices are expected to drop rendering robotic technology more widely available in the future. Extraperitoneal space can pose a small challenge for robot assistance due to the relative limited space available. The latter can reduce the range of robotic arms motion especially when a 4-arm da Vinci surgical system is used. Still, with proper modifications in trocar positioning extraperitoneal robot-assisted laparoscopic prostatectomy can offer a similar clinical outcome as the intraperitoneal approach rendering the selection of route a matter of surgeons preference based on his experience and expert [34, 35].

## 7. Conclusions

The lack of randomized controlled trials directly comparing different surgical approaches for radical prostatectomy precludes definite conclusions. Still, large series of patients subjected to LERP verify that the procedure offers, comparably to the open approach trifecta outcomes in addition to superior visualization of the operative field, less blood loss and better cosmesis. In matters of complications, the primary advantage of the extraperitoneal approach is the postoperative isolation of the surgical field outside peritoneal cavity, which sequesters potential postoperative urinary extravasation and enhances conservative healing. In turn, the latter appears to induce a higher incidence of lymphocele formation in cases of concomitant PLND which can be limited by peritoneal fenestration at the end of the procedure. Long and stiff learning curve associated with LERP affects both perioperative and oncologic outcomes in the initial case series. Thus, close mentoring supervision is advised. Finally, despite the inability of LERP to offer an extended PLND, the procedure still has a place in intermediate risk prostate cancer patients requiring lymph node sampling for staging purposes.
